# Diagnostic validation study of rapid urinary tract infection diagnosis kit at peripheral health facilities of West Bengal, India

**DOI:** 10.1038/s41598-023-49489-0

**Published:** 2024-01-02

**Authors:** Debjit Chakraborty, Falguni Debnath, Agniva Majumdar, Atreyi Chakrabarti, Monica Sharma, Kamini Walia, Alok Kumar Deb, Shanta Dutta

**Affiliations:** 1 ICMR-National Institute of Cholera and Enteric Diseases, P-33, CIT Road, Scheme-XM, Beliaghata, Kolkata, 10 India; 2 Department of Health and Family Welfare, Government of West Bengal, South 24 Pargana, India; 3 Epidemiology and Communicable Diseases Division, Indian Council of Medical Research, Ansari Nagar, New Delhi, India

**Keywords:** Public health, Infectious diseases, Health care, Medical research

## Abstract

Patients reporting to the outpatient departments of peripheral health care settings in India with symptoms of urinary tract infection (UTI) receive one or the other antibiotic before culture confirmation and out of the total culture confirmed UTI cases, in less than one third cases the prescribed antibiotics matches to the antibiotic sensitivity test result. Hence, in this study, an indigenous point-of-care (POCT) rapid diagnostic kit (Rapidogram) for UTI was validated against conventional urine culture and sensitivity to understand its possible applicability at peripheral health care settings. This cross-sectional study was conducted during November 2021 to June 2022 in OPDs of two peripheral hospitals. A sample size of 300 was calculated using prevalence of urinary tract infection (UTI) as 33% for sensitivity and specificity using Buderer’s formula. Urine specimens were collected following standard aseptic procedures from the recruited suspected UTI cases and transferred to laboratory maintaining the cold chain. The validation work up was done in two sections: lab validation and field validation. Out of 300 urine samples, 29 were found positive for the growth of UTI pathogen by both methods and 267 were found negative by both methods. Thus, the kit shows very high specificity (99.6%; 97.9–99.9%) and considerably high sensitivity (90.6%; 74.9–98.0%). We also observed higher PPV, NPV, test accuracy (> 96%). Diagnostic Odds Ratio and Youden index were respectively 2581 and 0.89. Clinical data showed that 44% of the suspected UTI cases were prescribed at least one antibiotic before urine test. Mostly they received Norfloxacin whereas the mostly identified organism *E.coli* was sensitive to Nitrofurantoin. In the context of absence of microbiology facility at peripheral setting and rampant empirical use of antibiotics in UTI, this highly specific and sensitive POCT for UTI may be used as it not only identifies the organism, also shows the antibiotic sensitivity pattern.

## Introduction

Bacterial urinary tract infections (UTIs) are one of the most common cause of hospital attendance globally and prevalent among all age groups, gender^1^. Females are more affected by UTIs, with an estimated lifetime risk of > 60%^2^. In 95% of the UTIs in women, Gram-negative bacteria are the causative agents. *Escherichia coli* alone causes 70% to 90% of it^1^. Diagnosis of UTI remains difficult in lower tiers of hospitals where microbiological testing facilities are unavailable. The hall mark of UTI is the presence of uropathogenic bacteria in urine, and culture of urine specimen is the reference standard method for the determination of clinically relevant bacteriuria. However, there is a dire need of more rapid diagnostic tool due to the long turnaround time needed for urine culture and antibiotic sensitivity result. The currently available rapid point-of-care methods for the detection of bacteriuria, including microscopy and test strips for detecting nitrite, suffers from poor sensitivity^3–5^. One of the studies reviewed 34 primary studies on evaluation of accuracy of nitrite test strips across settings and reported found a pooled mean sensitivity of 48%^6^.

In India, UTI is also one of the most commonly diagnosed infections in both hospitalized patients and community-residents. UTI is one of the most common conditions for antimicrobial use in older adults and this contributes heavily towards generation of multidrug resistant bacteria. UTIs in the community setting are further treated empirically in absence of the culture & sensitivity performing facility^7–12^. The most common pathogen associated with community-acquired UTI is *E.coli* followed by *Klebsiella*, *Proteus*, *Enterobacter*, *Staphylococcus saprophyticus*^13–17^ and have become resistant to several commonly used antibiotics^15,16^. Inappropriate clinical practices; lack diagnostic facility; mismanagement; overuse of antibiotics lack of awareness and self-medication have worsened the condition in developing counties like India. Hence to ensure evidence-based prescription of antibiotics, particularly in peripheral health care facilities where no microbiology facility is available, a point-of-care testing kit was developed by Sree Chitra Tirunal Institute for Medical Science & Technology. This locally produced point-of-care rapid kit (Rapidogram) has diagnostic sensitivity of 96% as reported by the manufacturer. Rapidogram was meant to detect presence of Gram negative bacteria in urine by changing colour as in primary care settings monomicrobial infection with Gram negative bacteria are most frequently encountered and treated empirically. We conducted the present study to validate the Rapidogram kit against conventional urine culture and sensitivity in suspected UTI patients at peripheral health facilities of a southern district of West Bengal, India.

## Methods

### Study setting

We conducted this cross-sectional study from November 2021 to June 2022 in two peripheral health facilities of West Bengal, a state in the eastern part of India. Indian public health care is a three tier system, primary, secondary and tertiary care from bottom up. Primary tier is present in rural and urban population close to community catering 30,000 to 50,000 population with primary healthcare services with minimum inpatient. Secondary hospitals have better facilities than primary but still do not have advanced facilities such as microbiology lab etc. They are located in one per subdivision (sub district). Tertiary hospitals are medical colleges with all department and regular facilities and mostly located in cities and act as referral hospitals. 80–85% patients of OPD and 70–75% patients of inpatient services are dealt by primary and secondary tier facilities as these are much more in number. As this kit was meant for rapid diagnosis of UTI at the peripheral health facility, we selected one Sub Divisional Hospital (SDH) and one Block Primary Health Centre (BPHC). The selection of district and health facilities was done through multistage simple random sampling. Participants were recruited from General OPD/ Medicine OPD/ Gynaecology OPD as applicable.

### Sample size calculation

We calculated the sample size at the required absolute precision level for sensitivity and specificity using Buderer’s formula^18^ and also using a nomogram^19^. Here we considered the sensitivity (SN) of Rapidogram to be 96% (0.96) as mentioned in their catalogue. We considered prevalence of 33% uropathogen among suspected UTI cases attending a tertiary hospital in Mumbai^12^ which was lower than a study in West Bengal^20^. Using the above values and formula for sensitivity, we arrived at a sample size of 279 and adding around 5% wastage rate, the final sample size was calculated to be 300.

### Recruitment and inclusion criterions

We used the case definition developed by Loeb et al.^10^, the minimum criteria for initiating antibiotics for UTI in residents without an indwelling urinary catheter which is as follows: Acute dysuria alone, OR Fever (> 37.9 °C or 1.5 °C increase above baseline temperature) and at least 1 of the following: New or Worsening (a) Urgency, (b) Frequency (c) Suprapubic pain, (d) Gross haematuria, (e) Costovertebral tenderness and (f) Urinary incontinence. As the study aimed to promote the rational use of antimicrobials in peripheral health settings, we found this diagnostic criteria as the most appropriate and simple one to be used by field Lab Technicians and primary care physicians.

Initial screening was done by facility medical officers as suspected UTI and then they referred to the study team comprising of field investigator and laboratory technician. Then the study team checked the eligibility as per prescription and patient history, took informed consent, and collected data and urine sample. In case of any doubt they discuss it with the clinician for final judgment. All investigators were previously trained by PI and Co-PI and facility clinicians and nurses were oriented about the study.

The kit validation workup was divided into two phases—an initial phase which involved laboratory validation followed by diagnostic validation on urine samples of suspected UTI cases.

### The point-of-care rapid kit (Rapidogram)

The UTI rapid diagnostic kit with antibiotic sensitivity was developed by Sree Chitra Tirunal Institute for Medical Sciences & Technology, Thiruvananthapuram, and the technology was transferred to Agappe Diagnostics Ltd which manufactured the kits. The Rapidogram kit detects significant bacteriuria directly from urine samples and also gives the antibiogram which can be read visually by observing changes in the colour of the test and control vials of the kit. The time required for the test is 3–6 h, thus saving time and enabling right treatment.

The Rapidogram kit is self-sufficient and contains everything needed for testing except an incubator at 37 °C. The test result is interpreted by observing the colour change in the control vial and the panel of 11 antibiotic vials—1. Ampicillin, 2. Amoxycillin + Clavulanic acid, 3. Cefuroxime, 4. Cefotaxime, 5. Ciprofloxacin, 6. Trimethoprim/ Sulphamethoxazole, 7. Gentamicin, 8. Amikacin, 9. Nalidixic acid, 10. Nitrofurantoin, 11. Norfloxacin. Sterile water (12 ml) is added to the sample preparation vial followed by addition of 3 ml of urine sample, the colour of the solution is checked and adjusted (if required) with “Reagent 1” and 1 ml of the solution is added to the control vial and each of the test vials. The vial marked control will give whether the patient has significant bacteriuria, and this can be read visually by observing change in colour from green to yellow. The antibiotic resistance is detected by the change in colour of the test vials having labels of the various antibiotics on incubation from green to yellow. The change in colour depends upon the growth of the pathogen. If the bacterium is resistant to the antibiotic used, the colour of the contents of the vial will turn from green to yellow. If the bacterium is sensitive to the antibiotic tested, then the original green colour will be retained.

The kit does not need sophisticated equipment and can be done in a primary health centre or the doctor’s clinic by anyone who can read and follow instructions. The doctor can have information on whether the patient has UTI and also which antibiotic can be used within 3 to 6 h.

### Laboratory validation

Accuracy, precision, repeatability, and limit of detection of the testing procedure were determined in a laboratory setting using serial dilutions of the ATCC strains of Gram-negative bacteria which are common pathogens of UTI—*Escherichia coli* ATCC 25922, *Klebsiella pneumoniae* ATCC 13883*, Proteus mirabilis* ATCC 25933 and *Pseudomonas aeruginosa* ATCC 27853).

#### Preparation of standard dilution

Pure colonies of the reference strains were inoculated in Luria–Bertani (LB) broth and kept at 37 °C for overnight incubation. The inoculum was further sub-cultured in LB broth and incubated for 5 h allowing the culture to achieve good active growth (i.e., mid-logarithmic phase). The broth was then centrifuged at 5000 rpm, washed twice with normal saline (NS), recentrifuged and suspended in NS. The turbidity was adjusted to 0.5 McFarland standard. Ten-fold serial dilutions were prepared from this inoculum.

#### Determination of CFU/ml

The standard dilutions of each reference strain were plated on Cystine lactose electrolyte deficient (CLED) agar in triplicate using the standard loop method (nichrome loop having diameter 4 mm–0.01 ml) and placed in a bacteriological incubator set at 37 °C. After overnight incubation, the CFU/ml was calculated from the average colony count/plate.

#### Testing in Rapidogram kit

A portion of the standard dilutions of each reference strain was simultaneously used in Rapidogram Kit as per kit instructions. The control vials were placed in a bacteriological incubator set at 37 °C and examined for colour change from green to yellow every hour until 6 h. The change in colour denoted significant bacteriuria and was analysed with the CFU/ml obtained from plating of the standards.

### Testing of clinical samples for diagnostic validation

Following written informed consent, cases were recruited at the study sites. Basic demographic and clinical information including antibiotic history was documented in a structured Case Record Form. We collected samples from middle of January 2022 and completed in middle of June 2022 till the 300 samples were reached. Sample collection from two facilities were done through consecutive sampling technique. Initially sample collection was relatively less due to low OPD attendance for COVID-19 Pandemic, but it improved in later months. First, we selected the district randomly from the list of districts in West Bengal state and once, the district was selected, we again chose the block primary hospital randomly from the list of those hospitals of that district. However, each district has only one sub divisional hospital, hence, we chose that.

#### Sample collection

The patients meeting the inclusion criterions were asked to obtain clean catch midstream urine samples in the properly labelled sterile wide-mouthed urine containers provided to them maintaining local aseptic measures^20,21^.

#### Sample transport

As we compared the Rapidogram test results with the conventional culture it was very important that the inoculation with the urine sample was done simultaneously in both the system. Since the chosen sites do not have a microbiology lab, this analysis was not possible at the sites. Moreover, the Rapidogram kit requires hourly monitoring until 6–8 h after inoculation which was not feasible at the peripheral sites (OPD based). In order to address these issues, urine samples were both kept temporarily at the site and transported under cold chain. soon after the end of daily OPD the samples were promptly kept in cool boxes maintained at a temperature of 4 °C until it was transported to the laboratory at ICMR-NICED maintaining cold chain. Each sample container was checked for leakage and cold chain maintenance on receipt. The samples were processed as soon as it reached the lab.

#### Testing in Rapidogram kit

A portion of the urine samples was used in Rapidogram Kit as per kit instructions. The vials were placed in a bacteriological incubator set at 37 °C and examined for colour change from green to yellow every hour until 6 h. The vial marked control gave whether the patient had significant bacteriuria. If the bacterium was resistant to the antibiotic used, the colour of the contents of the vial turned from green to yellow. The original green colour was retained if the bacterium was sensitive to the antibiotic tested.

#### Primary plating and isolation

The urine sample was thoroughly mixed and then plated on CLED agar using the standard loop method (nichrome loop having diameter 4 mm–0.01 ml) and placed in a bacteriological incubator set at 37 °C for 18 to 24 h incubation. Colony count was calculated to determine the presence of significant bacteriuria^21,22^.

#### Bacterial identification

If similar colonies were observed in numbers suggesting significant bacteriuria, a separate colony or a portion of apparently pure growth was subjected to Gram staining and microscopy followed by subculture in biochemical media for identification.

#### Antimicrobial susceptibility testing

AST was performed from all significant growth as per Kirby Bauer disc diffusion method using appropriately diluted inoculum of a pure growth^23^. Antimicrobial panel selection was based on those provided in the Rapidogram kit—Ampicillin, Amoxycillin + Clavulanic acid, Cephalexin, Cefuroxime, Cefotaxime, Ciprofloxacin, Trimethoprim + Sulphamethoxazole, Gentamicin, Amikacin, Nalidixic acid, Nitrofurantoin, Norfloxacin. After overnight incubation at 37 °C in bacteriological incubator, the zone diameters were interpreted as per CLSI guidelines^24^. The culture and AST report were communicated to the respective physician/ hospital authority.

#### Quality control

QC was ensured in the form of sterility and performance testing of each batch of prepared media, performance testing of reagents, QC for antibiotic discs and control strains as per CLSI guidelines^24^.

### Data analysis plan

The Rapidogram kit was evaluated against the reference Standard i.e. Conventional Urine Culture using the following statistics:

Discriminative ability of the diagnostic test was quantified by the measures of diagnostic accuracy as follows.

- sensitivity and specificity
- positive and negative predictive values (PPV, NPV)
- likelihood ratio
- Youden index^25^
- diagnostic odds ratio (DOR)^26^
- Accuracy

### Ethical consideration

We obtained approval of the protocol from the Institutional Ethics Committee of ICMR— NICED (No.A-1/2021-IEC dated May 13, 2021) We explained the objectives, risk & benefit of the study in clear, comprehensible vocabulary, in a local language, to all study participants before obtaining written consent/assent. Data security and Patient confidentiality were maintained throughout the study. OPD ticket registration number was utilized as anonymized identifier. The study was performed in accordance with the Declaration of Helsinki.

## Results

### Lab validation

During Lab Validation *Escherichia coli* ATCC 25922 caused change in colouration of the control vial for dilutions of minimum 1.30 × 105 CFU/ml while no change in colouration was observed for those dilutions of maximum 1.11 × 105 CFU/ml. *Klebsiella pneumoniae* ATCC 13883 caused change in colouration of the control vial for dilutions of minimum 0.38 × 10^5^ CFU/ml while no change in colouration was observed for those dilutions of maximum 0.32 × 10^5^ CFU/ml. *Proteus mirabilis* ATCC 25933 caused change in colouration of the control vial for dilutions of minimum 1.55 × 105 CFU/ml while no change in colouration was observed for those dilutions of maximum 1.42 × 105 CFU/ml. *Enterobacter aerogenes* ATCC 13048 caused change in colouration of the control vial for all dilutions of significant bacteriuria while no change in colouration for those dilutions which are not significant. *Pseudomonas aeruginosa* ATCC 27853 caused no change in the colouration of the control vial as mentioned by the manufacturer (Additional file 1).

### Field validation

We collected 300 urine samples from suspected UTI cases from two peripheral hospitals. The majority of patients were female (97.7%) and belonged to 15–35 years age group (59%). Major symptoms at presentation being dysuria (79.7%), urgency (60.7%) and frequency of Micturation (57.7%). Any antibiotic was prescribed before sample testing in case of 44% of patients and multiple antibiotics were prescribed in 2% of cases (Table 1). Norfloxacin (45%) was the antibiotic most commonly prescribed followed by Amoxycillin & Clavulinic Acid (39%). However, we collected the urine samples in that OPD only before they took the antibiotic. So prescription of antibiotic did not influence the result of urine samples.

**Table 1:** Distribution of suspected UTI cases as per demographic and clinical attribute.

n = 300	Number	%
Gender		
Female	293	97.7
Male	7	2.3
Age		
< 15 years	1	0.3
15–35 year	177	59.0
35–50 years	84	28.0
50 + years	38	12.7
Symptoms		
Dysuria present	239	79.7
Fever present	46	15.3
Haematuria present	9	3.0
Urgency	182	60.7
Frequency of micturation	173	57.7
Comorbidity		
Present	16	5.3
Antibiotic prescription		
Antibiotic prescription rate (empirical)	132	44.0
Multiple antibiotic prescription	6	2.0

Out of 300 urine samples, 29 were found positive for the growth of UTI pathogen by both methods and 267 were found negative by both methods. Thus, the Rapidogram kit shows very high specificity (99.6%; 97.9–99.9%) and considerably high sensitivity (90.6%; 74.9–98.0%). We observed higher PPV, NPV and test accuracy as well (> 96). Diagnostic Odds Ratio and Youden index were respectively 2581 and 0.89. We observed here a Likelihood Ratio + of 90 and Likelihood Ratio—of 0.1 (Table 2).

**Table 2:** Comparison of Rapidogram results with convention culture result for growth of UTI pathogen# with validation indicators. (n = 300).

Rapidogram result for growth	Culture result for growth		
	Positive	Negative	Total
Positive	29	1	30
Negative	3	267	270
Total	32	268	300

Validation indicators	Value (%)	95% CI (%)	
Sensitivity	90.6	(74.9–98.0)	
Specificity	99.6	(97.9–99.9)	
PPV	96.7	(82.8–99.9)	
NPV	98.9	(96.8–99.8)	
Accuracy	98.7	(96.6–99.6)	

We observed bacterial growth in 30 samples by Rapidogram Kit. The kit shows highest resistance rate for Ampicillin (70%) followed by Cefuroxime (43.3%). Maximum Sensitivity rate was evident for Amikacin (100%), Nitrofurantoin (90%) and Gentamicin (86.7%) (Table 3).

**Table 3:** Antibiotic sensitivity (S) and resistance (R ) pattern as observed in rapidogram method.

SL	Rapidogram Result (n = 30)	S	R	Total	S%	R%
1	Ampicillin	9	21	30	30.0	70.0
2	Amoxycillin/clavulinic acid	22	8	30	73.3	26.7
3	Amikacin	30	0	30	100.0	0.0
4	Cefotaxime	21	9	30	70.0	30.0
5	Cefuroxime	17	13	30	56.7	43.3
6	Ciprofloxacin	22	8	30	73.3	26.7
7	Gentamicin	26	4	30	86.7	13.3
8	Nalidixic Acid	22	8	30	73.3	26.7
9	Nitrofurantoin	27	3	30	90.0	10.0
10	Norfloxacin	23	7	30	76.7	23.3
11	Co-trimoxazole	24	6	30	80.0	20.0

By conventional culture, we identified organisms (both gram-negative and positive-bacteria) in 42 samples. Major organisms isolated included *E. coli, Klebsiella, Enterococcus, Staphylococcus *etc*.* We encountered two contaminated sample which we did not consider or sample producing mixed growth in culture. For *E. coli* highest antibiotic resistance rate was observed with Ampicillin (66.7%) followed by Cefuroxime (54%). The sensitivity pattern was highest for Aminoglycosides (Amikacin, Gentamicin) and Nitrofurantoin. Most commonly prescribed drugs both Norfloxacin and Amoxycillin/Clavulanate showed a sensitivity rate of 70%.

## Discussion

The studied indigenous rapid diagnostic kit was found highly specific (99%) and considerably sensitive (90%). The higher Diagnostic Odds Ratio and Youden index reflect the discriminative ability of the test. We observed here a Likelihood Ratio + of 90 and Likelihood Ratio—of 0.1. Ideally a good diagnostic tests should have a LR +  > 10 and LR- < 0.1. As UTI cases suffers from recurrent infections, appropriate antibiotic prescription is critical for their remission and prevention of development of antibiotic resistance. Different countries are also in an effort to develop such less time-consuming point of care diagnostic kit. A London based Microbiological Survey POCT kit which is simple and rapid was reported 100% specificity, 92% sensitivity and 97% accuracy^27^. An Indian study based from Bangalore attempted the Urine Dipstick method for rapid diagnosis of UTI cases at field level and found the method very useful reporting 95% and 73% specificity for Nitrite and Leucocyte Esterase respectively^28^. However, sensitivity of leucocyte esterase method was a concern and sensitivity were found to vary from 48.5 to 77% in several studies^29–33^. The present rapidogram kit was advantageous as it not only indicated presence of uropathogen but also showed antibiotic susceptibility pattern by colour changes which will surely help the clinician to select the first line antibiotics. We isolated *E. coli* as the major uropathogen and it was sensitive to nitrofurantoin. Similar findings were reported by other studies as well^28,34,35^. We witnessed that in absence of any urine investigation 44% of patients received any antibiotic and most commonly prescribed drug were Norfloxacin and Amoxycillin & Clavulinic Acid. However, Nitrofurantoin was found to be the oral antibiotic with highest sensitivity but not prescribed as a first line. This discrepancy is likely to happen in primary and secondary tier hospitals where there are no microbiology facilities Challenges of managing UTIs are just not restricted to large number of infections occuring every year, also the diagnosis was not straightforward always and require diagnostic facilities beyond clinical judgment^36^. In this study we have seen that in 44% of the cases empiric antibiotic has been prescribed and most of them are from watch category in WHO AWaRe classification. Nitrofurantoin is a drug of access category and also have shown sensitivity to the isolated organisms in most of the cases. This drug has also been recommended for empiric treatment in UTI in the guidelines released by Indian Council of Medical Research^37^. Left untreated or inadequately treated UTI can lead to sepsis in 30% cases particularly in geriatric population^38,39^.

In such context, considering the use of such POCT for diagnosis and also identification of antimicrobial sensitivity for UTI can help in rationalizing antimicrobial prescription and may result in early remission with decreased chance of development of resistance.

## Conclusion

The studied kit was a valid point of care rapid diagnostic kit in spite some limitation (meant only for UTI caused by Gram Negative bacteria and unable to detect Gram Positive organism) and thus may be considered for rolling out in primary and secondary level health care settings to promote rational antibiotic prescriptions, prevention of complication, minimize duration of illness and finally may contribute to containment of rising antimicrobial resistance.

### Supplementary Information

Supplementary Information 1.

Supplementary Information 2.

## Data Availability

Data are available with the PI of the project and can be made available on request to the corresponding author.
