# Supplementary material for: Diagnostic validation study of rapid urinary tract infection diagnosis kit at peripheral health facilities of West Bengal, India
Source: Sci Rep. 2024 Jan 2;14:297. doi: 10.1038/s41598-023-49489-0 (PMC10761820; doi:10.1038/s41598-023-49489-0)
Supplement: Supplementary file 1 — Supplementary Information 1. [file 41598_2023_49489_MOESM1_ESM.docx]

. Supplementary table

| ***Escherichia coli* ATCC 25922** | **Test #1** | **Test #2** | **Test #3** | **Test #4** | **Test #5** | **Test #6** |
| --- | --- | --- | --- | --- | --- | --- |
| Minimum CFU/ml causing change in colouration | 1.30 x 10^5^ | 1.32 x 10^5^ | 1.72 x 10^5^ | 2.40 x 10^5^ | 2.40 x 10^5^ | 1.70 x 10^5^ |
| Maximum CFU/ml causing no change in colouration | 0.33 x 10^5^ | 0.57 x 10^5^ | 0.55 x 10^5^ | 0.78 x 10^5^ | 0.89 x 10^5^ | 1.11 x 10^5^ |

*Escherichia coli* ATCC 25922 caused change in colouration of the control vial for dilutions of **minimum 1.30 x 10^5^ CFU/ml** while no change in colouration observed for those dilutions of **maximum 1.11 x 10^5^ CFU/ml**.

| ***Klebsiella pneumoniae***  **ATCC 13883** | **Test #1** | **Test #2** | **Test #3** | **Test #4** | **Test #5** |
| --- | --- | --- | --- | --- | --- |
| Minimum CFU/ml causing change in colouration | 0.38 x 10^5^ | 0.56 x 10^5^ | 0.48 x 10^5^ | 0.68 x 10^5^ | 0.88 x 10^5^ |
| Maximum CFU/ml causing no change in colouration | 0.11 x 10^5^ | 0.2 x 10^5^ | 0.17 x 10^5^ | 0.32 x 10^5^ | 0.29 x 10^5^ |

***Klebsiella pneumoniae* ATCC 13883** caused change in colouration of the control vial for dilutions of **minimum 0.38 x 10^5^ CFU/ml** while no change in colouration observed for those dilutions of **maximum 0.32 x 10^5^ CFU/ml**.

| ***Proteus mirabilis***  **ATCC 25933** | **Test #1** | **Test #2** | **Test #3** | **Test #4** | **Test #5** |
| --- | --- | --- | --- | --- | --- |
| Minimum CFU/ml causing change in colouration | 1.59 x 10^5^ | 1.86 x 10^5^ | 2.7 x 10^5^ | 2.66 x 10^5^ | 1.55 x 10^5^ |
| Maximum CFU/ml causing no change in colouration | 0.69 x 10^5^ | 0.61 x 10^5^ | 0.93 x 10^5^ | 0.96 x 10^5^ | 1.42 x 10^5^ |

***Proteus mirabilis* ATCC 25933** caused change in colouration of the control vial for dilutions of **minimum 1.55 x 10^5^ CFU/ml** while no change in colouration observed for those dilutions of **maximum 1.42 x 10^5^ CFU/ml**.

| ***Enterobacter aerogenes***  **ATCC 13048** | **Test #1** | **Test #2** | **Test #3** | **Test #4** | **Test #5** | **Test #6** |
| --- | --- | --- | --- | --- | --- | --- |
| Minimum CFU/ml causing change in colouration | 1.37 x 10^5^ | 2.15 x 10^5^ | 2.21 x 10^5^ | 2.28 x 10^5^ | 2.47 x 10^5^ | 1.07 x 10^5^ |
| Maximum CFU/ml causing no change in colouration | 0.45 x 10^5^ | 0.76 x 10^5^ | 0.83 x 10^5^ | 0.93 x 10^5^ | 0.77 x 10^5^ | 0.96 x 10^5^ |

***Enterobacter aerogenes* ATCC 13048** caused change in colouration of the control vial for all dilutions of significant bacteriuria while no change in colouration for those dilutions which are not significant.

***Pseudomonas aeruginosa* ATCC 27853** caused no change in colouration of the control vial as mentioned by the manufacturer.
