# Supplementary material for: Diagnostic validation study of rapid urinary tract infection diagnosis kit at peripheral health facilities of West Bengal, India
Source: Sci Rep. 2024 Jan 2;14:297. doi: 10.1038/s41598-023-49489-0 (PMC10761820; doi:10.1038/s41598-023-49489-0)
Supplement: Supplementary file 2 — Supplementary Information 2. [file 41598_2023_49489_MOESM2_ESM.docx]

Table 5: Antibiotic Sensitivity (S) and Resistance (R) Pattern as observed for E. coli in conventional antibiogram.

| SL | Conventional Antibiogram Result for E. coli(n=24) | S | R | Total | S% | R% |
| --- | --- | --- | --- | --- | --- | --- |
| 1 | Ampicillin | 8 | 16 | 24 | 33.3 | 66.7 |
| 2 | Amoxycillin/Clavulanic acid | 17 | 7 | 24 | 70.8 | 29.2 |
| 3 | Amikacin | 24 | 0 | 24 | 100.0 | 0.0 |
| 4 | Cefotaxime | 15 | 9 | 24 | 62.5 | 37.5 |
| 5 | Cefuroxime | 11 | 13 | 24 | 45.8 | 54.2 |
| 6 | Ciprofloxacin | 17 | 7 | 24 | 70.8 | 29.2 |
| 7 | Gentamicin | 20 | 4 | 24 | 83.3 | 16.7 |
| 8 | Nalidixic Acid | 16 | 8 | 24 | 66.7 | 33.3 |
| 9 | Nitrofurantoin | 21 | 3 | 24 | 87.5 | 12.5 |
| 10 | Norfloxacin | 17 | 7 | 24 | 70.8 | 29.2 |
| 11 | Co-trimoxazole | 19 | 5 | 24 | 79.2 | 20.8 |

For E. coli highest antibiotic resistance rate was observed with Ampicillin (66.7%) followed by Cefuroxime (54%). Sensitivity pattern was highest for Aminoglycosides (Amikacin, Gentamicin) and Nitrofurantoin. Most commonly prescribed drugs both Norfloxacin and Amoxycillin/Clavulunate showed a sensitivity rate of 70%.

Table 3: Comparison of Validation indicators of Rapidogram results with convention antibiogram result across different antibiotics. (n=29)

| **Validation Indicators (n= 29)** | **Ampicillin** | **Amoxycillin/Clavulanic acid** | **Amikacin** | **Cefotaxime** | **Cefuroxime** | **Ciprofloxacin** | **Gentamicin** | **Nalidixic Acid** | **Nitrofurantoin** | **Norfloxacin** | **Co-trimoxazole** |
| --- | --- | --- | --- | --- | --- | --- | --- | --- | --- | --- | --- |
| Sensitivity (%) | 87.0 | 66.7 | 0 | 75 | 100 | 50 | 33.3 | 42.1 | 0.0 | 60.0 | 50.0 |
| Specificity (%) | 83.3 | 90.0 | 100 | 100 | 100 | 100 | 91.3 | 100.0 | 89.3 | 94.7 | 84.0 |
| PPV (%) | 95.2 | 75.0 | 0 | 100 | 100 | 100 | 50.0 | 100.0 | 0.0 | 85.7 | 33.3 |
| NPV (%) | 62.5 | 85.7 | 96.6 | 85 | 100 | 62 | 84.0 | 47.6 | 96.2 | 81.8 | 91.3 |
| Accuracy (%) | 86.2 | 82.8 | 96.6 | 89.7 | 100 | 72 | 79.3 | 62.1 | 86.2 | 82.8 | 79.3 |

We observed growth of UTI Pathogens in both method for only 29 samples. Hence, we compared the antibiotic resistance pattern by both the methods and measured the validation indicators for detecting antibiotic resistance. Since the sample size is very low (29) and not calculated antibiotic wise we could not derive into any statistical conclusion. We have not calculated 95% Confidence Interval also as for such a low sample size CI will be wide and overlapping. However, this table only gives an idea about variability of the validation indicators across different antibiotics. The kit performance ranges from the worst for Nitrofurantoin to the best for Cefuroxime. A kit specificity higher than 90% for detecting antibiotic resistance was observed for Norfloxacin, Gentamicin and Amoxycillin/ Clavulanic Acid
